# Medial Prefrontal Cortex: Adding Value to Imagined Scenarios

**DOI:** 10.1162/jocn_a_00836

**Published:** 2015-06-04

**Authors:** Wen-Jing Lin, Aidan J. Horner, James A. Bisby, Neil Burgess

**Affiliations:** University College London

## Abstract

The medial prefrontal cortex (mPFC) is consistently implicated in the network supporting autobiographical memory. Whereas more posterior regions in this network have been related to specific processes, such as the generation of visuospatial imagery or the association of items and contexts, the functional contribution of the mPFC remains unclear. However, the involvement of mPFC in estimation of value during decision-making suggests that it might play a similar role in memory. We investigated whether mPFC activity reflects the subjective value of elements in imagined scenarios. Participants in an MRI scanner imagined scenarios comprising a spatial context, a physiological state of need (e.g., thirst), and two items that could be congruent (e.g., drink) or incongruent (e.g., food) with the state of need. Memory for the scenarios was tested outside the scanner. Our manipulation of subjective value by imagined need was verified by increased subjective ratings of value for congruent items and improved subsequent memory for them. Consistent with our hypothesis, fMRI signal in mPFC reflected the modulation of an item’s subjective value by the imagined physiological state, suggesting the mPFC selectively tracked subjective value within our imagination paradigm. Further analyses showed uncorrected effects in non-mPFC regions, including increased activity in the insula when imagining states of need, the caudate nucleus when imagining congruent items, and the anterior hippocampus/amygdala when imagining subsequently remembered items. We therefore provide evidence that the mPFC plays a role in constructing the subjective value of the components of imagined scenarios and thus potentially in reconstructing the value of components of autobiographical recollection.

## INTRODUCTION

Autobiographical memories (AMs) define who we are and depend on a network of brain regions including the hippocampus, parahippocampal gyrus, retrosplenial cortex, posterior parietal cortices, and medial prefrontal cortex (mPFC; e.g., Addis, Moscovitch, Crawley, & McAndrews, 2004; Piolino et al., 2004; Maguire, 2001; Nadel & Moscovitch, 1997). Research into the neural mechanisms underlying AM has focused on closely related concepts of imagery for spatial context (e.g., Burgess, Maguire, & O’Keefe, 2002), “scene construction” (e.g., Hassabis, Kumaran, & Maguire, 2007), “episodic future thinking” (e.g., Addis, Wong, & Schacter, 2007), “self-projection” (Buckner & Carroll, 2007), and item-to-context binding (Eichenbaum, Yonelinas, & Ranganath, 2007). In addition to the long-recognized hippocampal role in AM (Howard & Eichenbaum, 2013; Squire & Zola-Morgan, 1991; O’Keefe & Nadel, 1978; Scoville & Milner, 1957), this research has proposed specific functional roles for posterior brain regions. The parahippocampus, retrosplenial cortex, and the rest of Papez’s circuit have been ascribed roles in the generation of visuospatial imagery (Byrne, Becker, & Burgess, 2007), whereas medial-temporal regions have been implicated in storing items and context beyond the spatial domain (Eichenbaum et al., 2007). Furthermore, lateral parietal and prefrontal areas have been ascribed roles in attentional and working memory components of AM tasks (Johnson, Suzuki, & Rugg, 2013; Rugg & Vilberg, 2013; Cabeza, Ciaramelli, Olson, & Moscovitch, 2008; Simons et al., 2008; Wagner, Shannon, Kahn, & Buckner, 2005).

However, less is known regarding the functional role of mPFC in AM. In decision-making, mPFC responses are believed to represent the subjective value of chosen items relative to potential alternatives (Rushworth, Noonan, Boorman, Walton, & Behrens, 2011). Activity in mPFC is correlated with the value of the chosen item, irrespective of whether the items are food (Gross et al., 2014; Hare, Camerer, & Rangel, 2009), water (Bouret & Richmond, 2010), monetary reward (Nicolle et al., 2012; Boorman, Behrens, Woolrich, & Rushworth, 2009), physical action, engaging activities (Gross et al., 2014), or abstract figures (Glascher, Hampton, & O’Doherty, 2009). The mPFC is also associated with self-referential thought, including memory (Levine, 2004; Macrae, Moran, Heatherton, Banfield, & Kelley, 2004; Vogeley et al., 2004; Johnson et al., 2002; Gusnard, Akbudak, Shulman, & Raichle, 2001), leading to the recent suggestion that ventromedial pFC (vmPFC) helps to establish the personal value, affective quality, or significance of self-related information (Benoit, Szpunar, & Schacter, 2014; D’Argembeau, 2013; Lebreton et al., 2013).

Given the association between memory and imagery, it is interesting that imagery can interact with subjective value and can influence our motivation for satisfying basic needs, such as food consumption (Larson, Redden, & Elder, 2014; Morewedge, Huh, & Vosgerau, 2010). In addition, imagining future scenarios can influence decision-making by changing the subjective value of choices (Lebreton et al., 2013; Benoit, Gilbert, & Burgess, 2011; Peters & Büchel, 2010). Thus, imagining oneself in a hungry state may raise the subjective value of food items. Conversely, human memory can be influenced by the value or motivational salience of the to-be-remembered stimuli (Erwin & Ferguson, 1979). For instance, fasting people have enhanced memory for food pictures (Morris & Dolan, 2001). Thus, we infer that memory for items could also be modulated by their value in imagined scenarios.

Building on these previous studies, we hypothesized that mPFC plays a role in AM and self-related imagery by providing the subjective value of elements of a scene—a function not ascribed to more posterior parts of the AM network. To test this hypothesis, we designed a paradigm in which the subjective value of items within imagined scenarios could be manipulated experimentally during fMRI. We required participants to imagine being in a current context and state (as opposed to imaging a future scenario, see Benoit et al., 2014) and subsequently imagine seeing, but importantly not consuming (cf. Gross et al., 2014), objects that were congruent or incongruent with the imagined state of need. We reasoned that the imagined current state of need would modulate the subjective value of the unconsumed objects and that mPFC activity would correlate with this state-modulated subjective value.

## METHODS

### Participants

Twenty right-handed participants were recruited from the University College London student population. One did not finish the task, so the data reported here concern the remaining 19 participants (12 women). The mean age of the remaining participants was 21.7 years (*SD* = 2.68, range = 19–27). All participants gave written informed consent to participate, in accordance with the local ethics committee (1825/003). One participant did not complete the postscan memory task, so the results from the memory analyses are based on 18 participants.

### Stimuli and Design

Four different physiological states of need were used: thirst, coldness, hunger, and tiredness. A neutral state was used as a baseline condition (instruction for neutral state: Imagine you are just fine. You are not in any state of need but just in an ordinary condition.). Twelve spatial contexts were used: beach, kitchen, desert, fields, classroom, airplane, forest, office, library, playground, church, and ship. These were included to make the imagined scenarios more realistic and because, without instruction, participants would be likely to imagine uncontrolled backgrounds to facilitate imagery. There were 60 state–context combinations, with each appearing only once during the 60 trials of the imagery task.

Pictures from four categories were used as items; each category contained items that were usually used to satisfy one of the four physiological states of need. The first category contained water, juice, beer, and other beverages used to quench thirst. The second category contained items that were able to be used to help people resist cold weather, such as fireplace, hot drink, and winter clothes. Another category contained food, and the final category contained items used for taking a rest or relieving tiredness included a bed, couch, bathtub, and so on. There were 180 item pictures in total, consisting of 45 pictures per category. Among these pictures, 120 appeared in the imagery task and another 60 served as new items during an old–new recognition test. The assignment of pictures to old items and new items was counterbalanced across participants. All pictures were obtained from FreeDigital-Photos.net (www.freedigitalphotos.net/).

**Figure 1:**
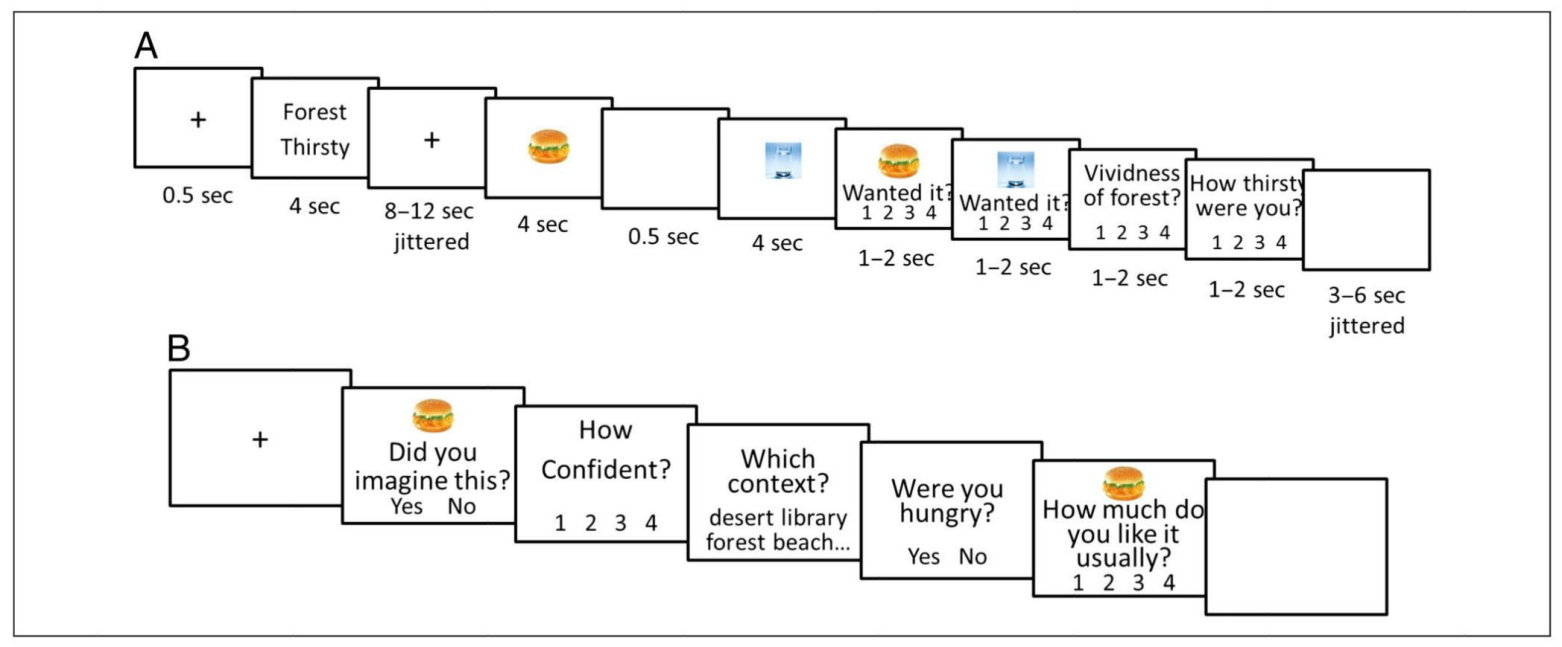
Procedure of the imagery task (A) and the memory task (B).

In the imagery task, each trial contained one state–context combination presented as cue words and also two item pictures (see Figure 1A for an example of trial presentation order). The relationship between the participant’s current imagined state and each item picture during a single trial could either be congruent or incongruent. For a congruent item, the type of item presented would meet the participant’s current need created by the imagined state. For instance, a food picture would be classified as congruent if the state was hunger, but incongruent if the state was tired, cold, or thirsty. Note that “incongruent” items were irrelevant rather than opposite to the current state of need. Ambiguous items were never used as “incongruent items” (e.g., a hot drink was not used in thirst trials). From the two item pictures, sequentially presented during each trial, either item could be congruent or incongruent with the current state. This provided four possible combinations: congruent–congruent, incongruent–incongruent, congruent–incongruent, and incongruent–congruent. Importantly, all four combinations of items occurred in pseudorandom order across trials, allowing us to identify the effects of an individual items’ subjective value, as modulated by its congruency with the imagined state. Among the 120 item pictures presented during the imagery task, 24 served as neutral pictures as they occurred in a neutral state. An alternative would be to use items unrelated to any of the physiological states, but such items would be intrinsically different to the congruent items in the study. The remaining 96 pictures were equally assigned as congruent or incongruent items.

### Procedure

#### Imagery Task

Participants were provided with task instructions before scanning and completed a number of practice trials outside the scanner. The entire imagery task, consisting of 60 imagery trials, was equally divided into two sessions, and scanning lasted for 1 hr in total, including acquisition of a structural scan. See Figure 1A for an illustration of stimulus presentation for the imagery task. Each trial began with a fixation cross at the center of the screen, which was replaced by a pair of state–context cue words after 0.5 sec. Participants were instructed to vividly imagine the context and state according to the cue words provided. The state–context cue words were presented for 4 sec, and then a fixation cross appeared again (for 8–12 sec, jittered), during which the participants were instructed to continue imagining. Next, two pictures were presented sequentially, each for 4 sec separated by a 0.5-sec blank screen. Participants were required to incorporate each presented item into their imagined scenario during the trial. Participants were explicitly instructed to not imagine consuming these items to satisfy their imagined state and its associated need. For example, they were required to imagine seeing (but not consuming) a chicken burger in a forest while they were thirsty (as in Figure 1A). After a further blank screen (1–4 sec, jittered), participants made four simple ratings, one at a time. The first two ratings asked participants to rate how much they had wanted each item when they initially saw it during the trial. The last two separately rated how vividly they had imagined the current state and context. All ratings used a 4-point scale (1 = *not at all*, 4 = *very much*). Each trial ended with a final blank screen (3–6 sec, jittered). Visual stimuli were presented by MATLAB (The MathWorks, Natick, MA) and COGENT 2000 toolbox (www.vislab.ucl.ac.uk/cogent.php).

#### Memory Task

The memory task took place outside the scanner after the imagery task was completed. Each trial consisted of a 500-msec fixation cross followed by a picture of an item, and participants were required to judge whether the picture had been presented in the imagery task or not (i.e., old/new item recognition judgment) and how confident they were of their answer (Figure 1B shows an illustration of the memory task). If participants answered “new,” participants were then asked how much they like that item in their daily lives. If an item was judged “old,” two further source memory questions were presented to the participant to test memory for the associated state and context. To test state, one of the state words (hunger, thirst, tired, cold, or neutral) was presented, and participants judged whether that state was the one they had been asked to imagine when the recognized item picture had appeared in the imagery task. The correct answer was yes for 50% of trials, and within these trials, 40% of the state words were congruent with the tested item, 40% were incongruent, and 20% were neutral. For the context source memory test, all 12 of the contexts were listed to allow participants to select the one which had accompanied the recognized item picture. The trial ended with the daily subjective rating. There were 180 memory trials in total (120 with “old” items and 60 with “new” items). Twelve alternative forced choice is an efficient way to test memory for the spatial context of an item’s presentation but could not be used to test memory for the physiological state, because a simple strategy of guessing the congruent state would artificially inflate performance (e.g., choosing “thirst” when presented with a drink). In this situation, choosing a congruent state would be correct in 40% of trials, a neutral state would be correct in 20% of trials, and the three incongruent states would be correct in 13% of trials. To avoid this, we tested participants with yes/no cued recognition of a single state that was chosen to be correct 50% of the time, irrespective of its congruence with the item.

#### fMRI Data Acquisition and Preprocessing

Functional imaging was performed on a 3T scanner (Siemens TIM Trio, Siemens, Berlin, Germany) during the imagery task. The functional data were acquired with a gradient-echo EPI sequence (repetition time = 3.36 sec, echo time = 30 msec, flip angle = 90°, resolution = 3 × 3 × 3 mm, 64 × 74, 48 slices per volume). The total number of volumes in each run varied across participants because of the variation of RT for each rating (the mean number of volumes was 332 per session). A high-resolution T1-weighted 3-D structural image (1 mm^3^) was acquired after two sessions of functional scans. A double-echo FLASH fieldmap sequence was also recorded.

Functional images were processed and analyzed with SPM8 (Wellcome Trust Centre for Neuroimaging, London, UK, www.fil.ion.ucl.ac.uk/spm/software/spm8/). The first five volumes of each scan were discarded for T1 equilibration. Preprocessing procedures included bias correction, realignment, unwarping, coregistration, slice timing correction, and normalization to the MNI template using the Dartel toolbox. EPI images were smoothed with an isotropic 8 mm FWHM Gaussian kernel. One of the participant’s fieldmap scan was not collected, so the unwarping procedure was skipped in their data.

#### Data Analysis

The preprocessed functional images were analyzed with general linear models (GLMs). We estimated five GLMs for different purposes. All GLMs included six movement regressors for each session, estimated during realignment, as well as two further regressors modeling each session. On the basis of our strong a priori hypothesis about the mPFC and vmPFC, we performed small-volume correction (SVC) within a combined anatomical mask of these regions: bilateral mPFC and vmPFC (volume ~ 53,493 mm^3^). This mask was derived from the AAL atlas (Tzourio-Mazoyer et al., 2002), as implemented in the WFU PickAtlas Tool (Maldjian, Laurienti, Kraft, & Burdette, 2003). This mask contained superior frontal gyrus, medial frontal gyrus, anterior cingulate, and cingulate gyrus. Within this small volume, we report effects that survive *p* < .05 FWE correction. For completeness, we also report effects at *p* < .001 uncorrected across the whole brain; however, caution is needed in interpretation of these effects.

The first model (GLM1) was a parametric modulation analysis, searching for regions that correlated with the subjective value of an item during imagined states of need. The first-level model contained seven regressors per session: (1) imagining a state of need, (2) imagining a neutral state, (3) imagining an item in a state of need, (4) a parametric modulator of the item regressor based on the participant’s subjective value of each item, (5) imagining an item in a neutral state, (6) intertrial interval (ITI) periods, and (7) key presses. Trial periods were modeled with a boxcar function for the entire length of each period (e.g., the 4 sec of imaging an item), convolved with the canonical hemodynamic response function. The second-level analysis was a one-sample *t* test on the parameter estimates from the parametric modulator (Regressor 4) averaged across the two sessions. For the parametric modulation, we used the subjective rating of each item when imagined in the state of need of the current trial minus the subjective rating of the item in the participant’s daily life, given after the scanning session. This calculation allowed us to control for variations in the participants’ baseline preference for the various items. The range of these normalized subjective ratings was from −3 to 3.

The second model (GLM2) was used for comparing imagination of congruent items versus incongruent items (given that the first GLM collapsed across these conditions to maximize power in our parametric modulation analysis) and also for comparing imagining states of needs versus neutral states. This model included seven regressors per session: (1) imagining a state of need, (2) imagining a neutral state, (3) imagining a congruent item in a state of need, (4) imagining an incongruent item in a state of need, (5) imagining an item in a neutral state, (6) ITI periods, and (7) key presses. Parameter estimates for regressors (1) to (4) were averaged across the two sessions and entered into a second-level model. A separate regressor was also included for each individual subject that consisted of a “1” for each condition for that specific participant (i.e., subject effects). A third model (GLM3) aimed to test the subsequent memory effect for imagined items. The model was similar to GLM1 but replaced the subjective value parametric modulator with a modulator based on subsequent memory. The model included six regressors per session: (1) imagining a state of need, (2) imagining a neutral state, (3) imagining an item (in either a state of need or neutral state), (4) a parametric modulator of the previous regressor based on subsequent memory for the item, (5) ITI periods, and (6) key presses. Note that the parametric modulator for subjective value was applied to item imagination during a state of need, not during neural states, as we were specifically interested in how states of need modulated subjective value. The parametric modulator for subsequent memory was applied to all item imagination trials (including neutral states) to maximize power. Subsequent memory was parameterized as a transformed confidence rating to maximize sensitivity. Participants’ 1–4 confidence ratings for old and new items at test were transformed into a measure of successful memory performance by combining ratings for item “hits” with negative ratings for item “misses” (e.g., a “miss” given a confidence rating of 4 would become −4 in the parametric modulator). The second-level analysis was a one-sample *t* test on the parameter estimates from the parametric modulator (Regressor 4) averaged across the two sessions.

The final two models (GLM4 and GLM5) aimed to test the subsequent memory effect for the state of need (GLM4) and the context (GLM5) in which items were imagined (i.e., two types of source memory). GLM4 contained seven regressors per session: (1) imagining a state of need, (2) imagining a neutral state, (3) item imagination trials for which the item and state of need are subsequently remembered, (4) item imagination trials for which the item but not the state is remembered, (5) item imagination trials for which the item is not remembered, (6) ITI periods, and (7) key presses. GLM5 was similar to GLM4 but split item imagination trials (Regressors 3–5) by whether the context (rather than the state) was remembered. Second-level models for each GLM were paired *t* tests comparing either state or context hits versus misses (Regressors 3 and 4) averaged across the two sessions.

Note that we built separate GLMs for each analysis of interest. This was due to the overlapping nature of certain regressors. In particular, the categorical congruent versus incongruent contrast correlated with the related, but more sensitive, item-by-item parametric modulation of value by state. Furthermore, the parametric modulators relating to subsequent memory and subjective value were also correlated. Despite the overlapping nature of these regressors of interest, our separate GLMs revealed distinct patterns of activity.

## RESULTS

### Behavioral Results

#### The Subjective Value of Items in Imagery

To demonstrate that our manipulation of imagined state worked, a three-way repeated-measure ANOVA with Situation (two levels: everyday rating and rating during imagery), Rating (1–4), and category (congruent, incongruent, and neutral) was performed. The three-way interaction was significant (*F*(6, 102) = 18.70, *p* < .001), so we performed further analyses that revealed that the distributions of ratings differed between categories for ratings during imagery (Rating × Category, *F*(6, 102) = 27.40, *p* < .001), but not for everyday ratings (Rating × Category, *F*(6, 102) = .66, *p* = .68). Thus, it was only when participants imagined being in a specific state of need that the subjective value of the objects differed between our “congruent” and “incongruent” conditions. Table 1 shows that a greater proportion of congruent items had positive subjective value (controlling for baseline value, i.e., rating of imagined value—everyday rating; 39.67%) whereas most incongruent items had negative subjective values (60.25%). This suggests that our participants indeed followed the instruction to imagine the assigned state of need and that those imagined states influenced the subjective value of the item on that trial.

We also carried out a two-way repeated-measure ANOVA with Congruency between the state question word and item (congruent and incongruent) and Rating (1–4) as within-subject variables to test whether the preceding state question might bias ratings (e.g., “hungry” increasing ratings for food items). There was no significant interaction between Congruency and Rating (*F*(3, 51) = .40; *p* = .75), suggesting that the everyday value ratings were not influenced by the preceding source memory questions.

#### Old–New Recognition

**Figure 2:**
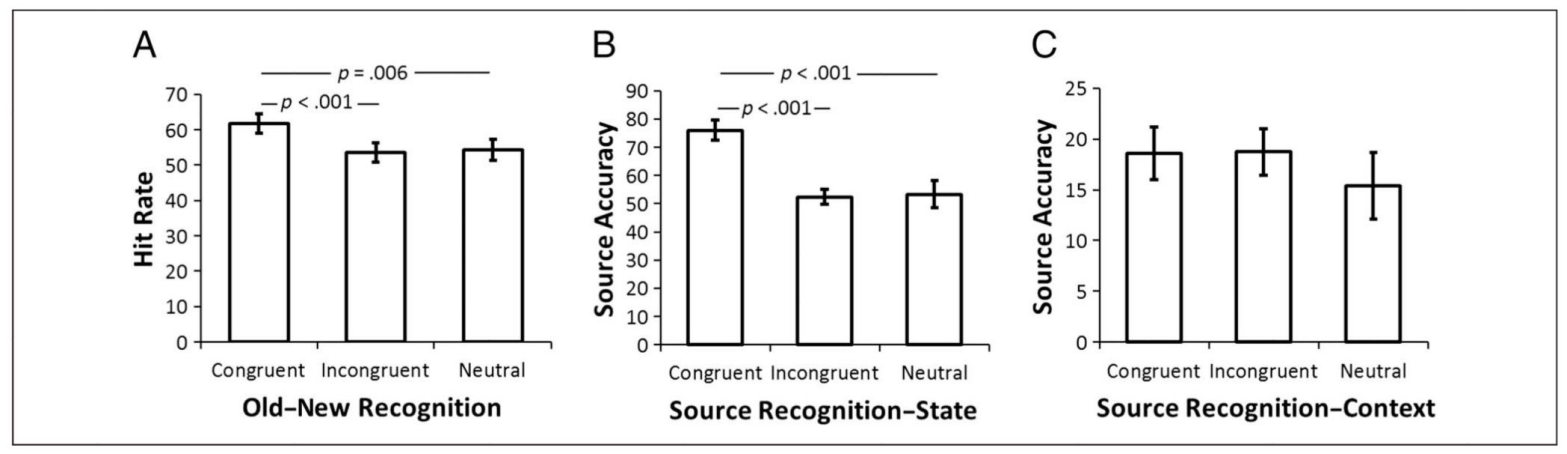
Behavioral results for the memory task. (A) Mean values of hit rate in the item recognition memory task. (B and C) Mean performance in the source recognition task for the state of need (B) and the spatial context (C). Error bars represent ±1 *SEM*.

A one-way repeated-measure ANOVA across Congruency (congruent, incongruent, and neutral) was carried out to test for differences in hit rate among different categories of items. The results revealed a significant main effect of congruency (*F*(2, 34) = 9.01, *p* < .001; see Figure 2A for memory performance). Pairwise comparisons showed that hit rate was higher for congruent items than for incongruent (*t*(17) = 5.16, *p* < .001) and neutral (*t*(17) = 3.14, *p* = .006) items. However, there was no significant difference between incongruent and neutral items (*t*(17) = .35, *p* = .73). This result suggests that participants had better memory for items that were able to fulfill their needs in the imagined state. Participants showed a high correct rejection rate for new items (87%). Table 2 shows confidence ratings across all responses.

For completeness, we checked whether our results varied with the order in which items were presented within a trial. We ran a two-way repeated-measure ANOVA with Order of presentation (two levels: first or second) and Category (three levels: congruent, incongruent, and neutral) as within-subject factors on the subjective ratings and subsequent memory scores. The results show that the order of presentation during encoding did not affect item memory (Order, *F*(1, 17) = .20, *p* = .66; Category, *F*(2, 34) = 9.22, *p* = .001; Order × Category, *F*(2, 34) = 1.89, *p* = .17), and there was a nonsignificant trend toward lower ratings for the first item versus the second item (Order × Category, *F*(2, 34) = 1.05, *p* = .36; order, *F*(1, 17) = 3.92, *p* = .06).

#### Source Memory

Source memory performance for correctly associating the imagined state with the recognized item was analyzed using a one-way ANOVA across levels of Congruency. We found a significant main effect of Item congruency (*F*(2, 34) = 17.30, *p* < .001). Pairwise comparisons showed that the conditional state source performance hit rate (% correct source memory for the state associated with items correctly recognized as “old”) for congruent items was significantly higher than for incongruent items (*t*(17) = 6.16, *p* < .001) and neutral items (*t*(17) = 5.44, *p* < .001), whereas there was no significant difference between the latter two categories (*t*(17) = .17, *p* = .864; see Figure 2B). Although participants showed a response bias toward accepting the state (answering “yes”) when it was congruent (55.6% responses were yes) or neutral (54.2% yes) relative to the item and “no” when it was incongruent (41% responses for incongruent items were no), this response bias could not account for our results (the correct proportion of “yes” responses being 50% in both cases).

Analysis of source memory performance for the imagined spatial context (e.g., “beach”) within the recognized item showed no significant main effect of Item congruency (*F*(2, 34) = .889, *p* = .42; see Figure 2C for context source memory performance). It is possible that this reflects the irrelevance of spatial context to the subjective ratings that the participants are required to give on each trial or that any small effects of congruency on context–source memory were obscured by low levels of performance (chance = 8%) although performance was above chance in each category (congruent: *t*(17) = 3.96, *p* = .001; incongruent: *t*(17) = 4.48, *p* < .001; neutral: *t*(17) = 2.14, *p* = .047).

In general, behavioral results supported our prediction. Subjective values of items support the validity of our imagined need paradigm. We also saw greater recognition performance for congruent than incongruent items and better memory for the imagined state of congruent than incongruent items. Thus, we observed better memory performance for items when their value was congruent with the imagined state.

### fMRI Results

#### Subjective Value of Items in Imagery (GLM1)

**Figure 3:**
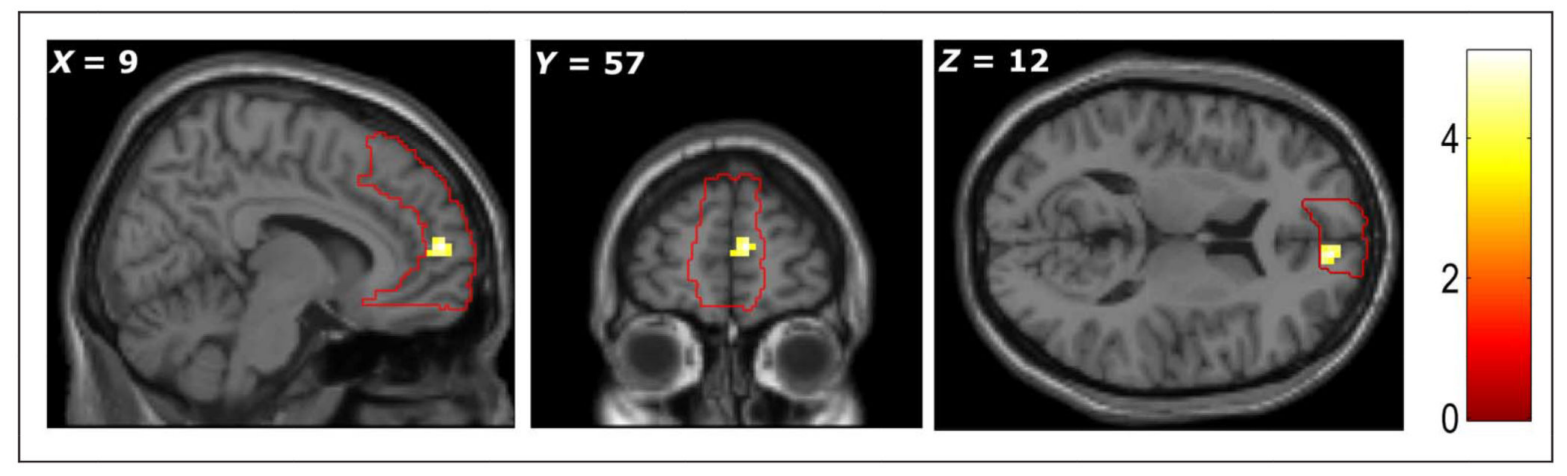
The activation of mPFC during imagination of an object during a state of need varied according to the extent to which the subjective value of item was modulated by the imagined state of need. All peaks significant at *p* < .001, uncorrected (color bar indicates *t* statistic). (The red line depicts the area of mPFC mask used in SVC analysis.

First, we focused on the main prediction of our study: that the subjective value of items in imagined scenarios would correlate with the BOLD response in the mPFC. To isolate imagined value from differences in the intrinsic values of the items used, we calculated the participant’s subjective value for the item when imagining it in the current state of need minus their subjective value for the same item in their daily life. This parametric modulator revealed an effect in the mPFC (+9, +57, +12, *Z* = 3.98; *p* < .05 FWE SVC). We therefore provide evidence that mPFC represents the values of elements in imagined scenarios, controlling for variations in their intrinsic value in other situations (Figure 3).

Given the complexity of our imagination task, it is important to rule out other explanations for our main mPFC result. This is particularly important given the overlapping nature of certain experimental factors (see Methods). In short, none of our subsequent analyses showed an effect in mPFC, even at a lenient *p* < .001 uncorrected threshold. However, these analyses did reveal effects in other regions at this threshold. We report these results for completeness but note that they should be treated with caution given that they do not survive correction for multiple comparisons.

#### Imagining States of Need and Item Congruency with Need (GLM2)

**Figure 4:**
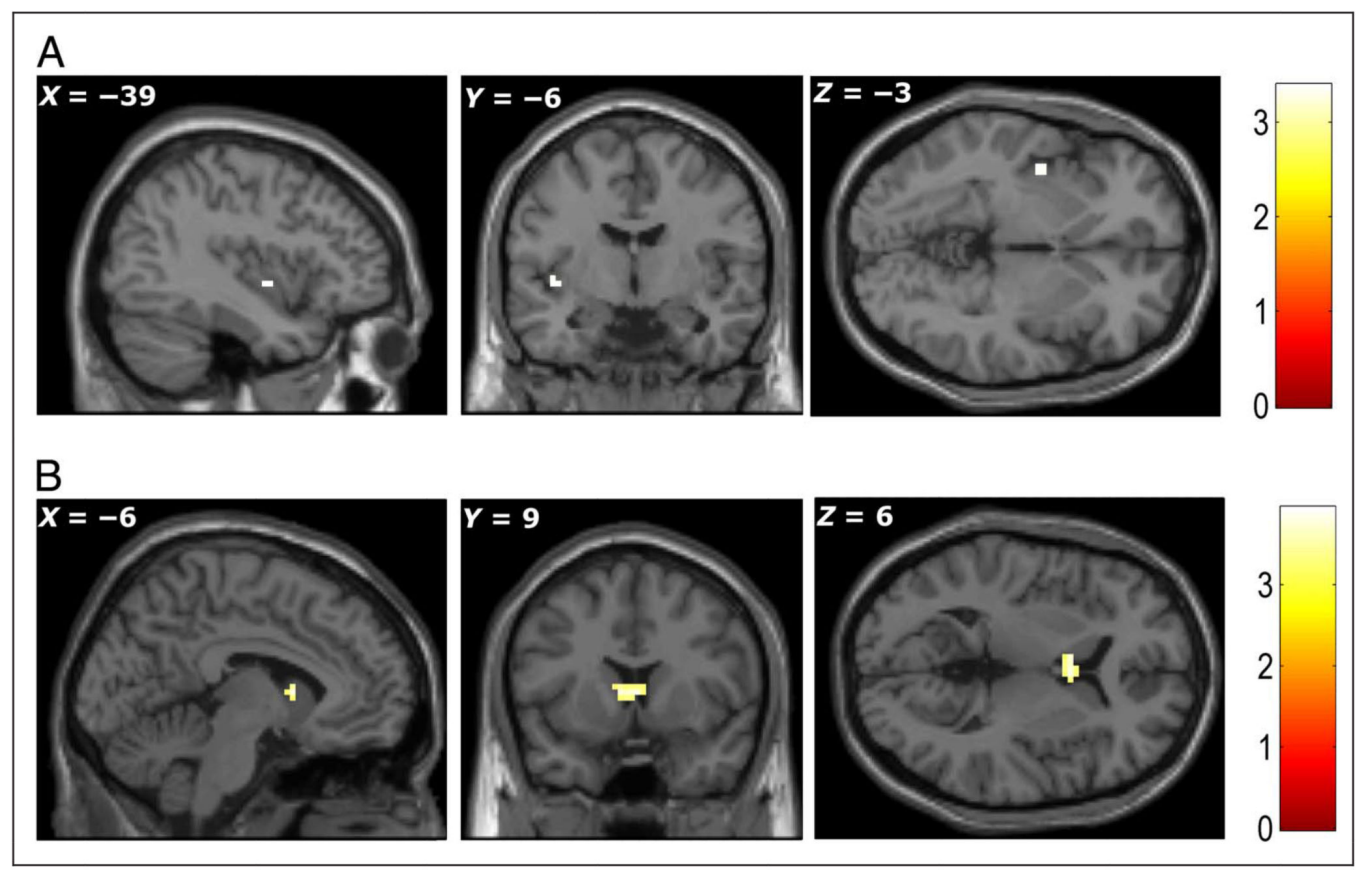
(A) Bilateral insula showed higher activation when participants were imagining states of need compared to imagining the neutral state. (B) The caudate showed greater activation for imagining a state-congruent item than a state-incongruent item. All peaks significant at *p* < .001, uncorrected (color bars indicate *t* statistic)

Compared with imagination of a neutral state, imagination of states of physiological need showed greater activation in bilateral insula (MNI coordinates of peak activations: −39, −6, −3, *Z* = 3.27; +45, +15, +3, *Z* = 3.15; *p* < .001, uncorrected; Figure 4A). By contrasting imagery for congruent versus incongruent items, we identified a region in the basal ganglia—the caudate nucleus (+3, +9, +6, *Z* = 3.60; −6, +9, +6, *Z* = 3.56, *p* < .001, uncorrected; Figure 4B). Because congruent items had higher subjective value than incongruent ones, we also carried out an SVC analysis for the congruent–incongruent contrast in the mPFC ROI but found no significant effect.

We also investigated whether the fMRI correlates of an item’s value or state congruency varied between the first and second item, finding a nonsignificant trend toward a greater effect of state congruency for the first versus second item in the vmPFC (−3, 33, −12; *p* = .083 FWE SVC). However, these could not influence the findings themselves, as our manipulation of state congruency was counterbalanced across items.

#### Subsequent Memory Effects (GLM3)

**Figure 5:**
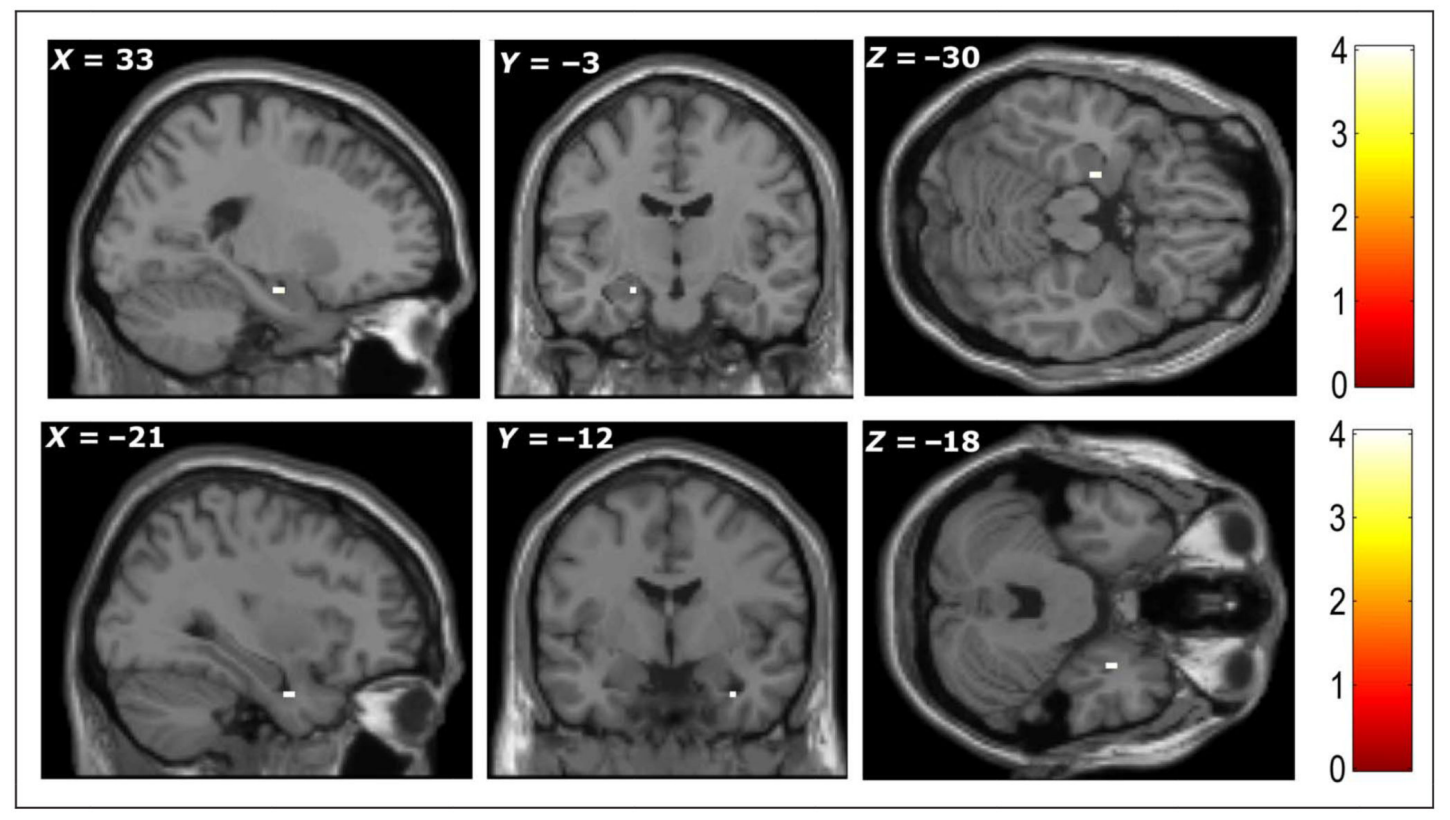
Greater activity was seen during encoding in left hippocampus (top row) and right amygdala (bottom row) for items that were subsequently correctly recognized with high confidence compared to subsequently nonrecognized items. All peaks significant at *p* < .001, uncorrected (color bars indicate *t* statistic).

This parametric modulation analysis showed that BOLD signal in the right amygdala (+33, −3, −30; *Z* = 3.27) and left anterior hippocampus (−21, −12, −18; *Z* = 3.33), when participants were imagining items, were significantly correlated with participants’ subsequent memory (*p* < .001, uncorrected; Figure 5). Note that our subsequent memory modulator combined categorical subsequent memory status (i.e., hits and misses) with subjective confidence, revealing linear increases in BOLD response from −4 (high confidence misses) to +4 (high confidence hits). No other significant activity was revealed in this analysis.

No significant activations were found corresponding to subsequent source memory effects for state (GLM4) or for context (GLM5), that is, the comparisons of imagery for items that became source hits versus source misses. This may reflect a lack of power, given the relatively low trial numbers in specific conditions (i.e., source misses for state), and the absence of a parametric measure like the confidence ratings used for item memory.

## DISCUSSION

We were interested in the potential role of mPFC in contributing subjective value to the contents of imagery. Our paradigm provides a way to measure this by manipulating subjective value of imagined items with respect to imagined physiological need. The behavioral results suggest that the manipulation was valid, and the imaging results support the hypothesis that mPFC activity reflects the subjective value of elements in imagined scenarios.

The manipulation of imagined need succeeded in altering the subjective value of elements within imagined scenarios in that participants indicated higher ratings for items congruent with (i.e., likely to satisfy) the state of need. Subsequent recognition memory for items also supports the success of our manipulation. Items that were able to fulfill people’s imaginary needs showed greater subsequent memory, both in being better recognized and being better associated to the state of need in which they were presented. This could be because to imagine a congruent item in the imagined scenario is more consistent with our daily life experiences and this enabled participants to have a richer imagination. Similarly, congruent items might fit more readily into a preexisting “schema” allowing for a more rapid integration of the item and imagined state (Tse et al., 2007; Bransford & Johnson, 1972; Bartlett, 1932). Equally, congruent items might have been better remembered because more valuable scenarios tend to be more strongly represented in memory-related areas (Lebreton, Jorge, Michel, Thirion, & Pessiglione, 2009; Wittmann et al., 2005).

The instruction to imagine states of physiological need was accompanied by increased activity in the insula compared to neutral states, albeit at an uncorrected threshold. This would be consistent with studies showing insular activation corresponding to interoception of actual physiological states (Craig, 2003), including thermo sensation (Craig, Chen, Bandy, & Reiman, 2000) and hunger (Tataranni et al., 1999). One might wonder whether people are able to imagine themselves in different physiological states, because physiological states are not usually thought to be under cognitive control. However, involuntary physiological signs can be influenced by imagination, for example, pupil dilation can be affected by imagining dark or light environments (Laeng & Sulutvedt, 2014).

We were interested in the process by which subjective value is afforded to an item within an imagined scenario. To investigate this, we looked for an fMRI signal matching the modulation of an item’s subjective value by the imagined state of need, that is, a regressor formed from the subjective rating of the item when imagined as part of a specific scenario minus the subjective rating of that item in daily life. We found activity following this pattern in mPFC, both in a more superior region and the ventral region of mPFC (albeit at an uncorrected threshold for the latter region; see Table 3). This is consistent with our hypothesis for the role of mPFC in imagery. Thus, beyond the representation of the subjective value of choices in decision-making, the mPFC may also play a role in representing the value of items in imagined scenarios more generally. This more general role might begin to explain its involvement in AM retrieval or episodic future thinking, as well as tasks with an implied component of choice such as planning. Indeed, mPFC activation has been seen together with hippocampal activation during the imagination of rewarding future situations in a decision task (Lebreton et al., 2013).

In general, congruent items were rated as more valuable than incongruent ones. Congruent items might be valuable because of their utility in a specific context (i.e., a congruent state; Hare, Malmaud, & Rangel, 2011) or because congruent items are more self-relevant in a congruent state (D’Argembeau, 2013). Could the results we observed in mPFC be caused by semantic congruency effect? To examine the effect of semantic congruency itself, we simply compared the imagination of explicitly congruent or incongruent items, finding activity in the caudate nucleus (but not in mPFC, where the difference in activity was some way below threshold, at *p* = .06 uncorrected). Thus, there is little support for a semantic interpretation of the mPFC activity we observed. The representation of the combined scenario may involve the striatum, via increased consolidation of the congruent state–item association, consistent with some rodent studies of consolidation (Pennartz et al., 2004). Alternatively, the striatal activation may reflect the involvement of these areas in reward-related processing (e.g., Knutson, Rick, Wimmer, Prelec, & Loewenstein, 2007), in the sense that the imagined interaction with the congruent item seems more rewarding in nature (although we forbade imagined consummation of items).

The behavioral results demonstrate a higher recognition rate for congruent items. This memory effect could relate to schema theory: perhaps the encoding of new information (i.e., a congruent item) benefits from being congruent rather than incongruent with the existing scenario. The mPFC has been implicated in incorporating new information into existing knowledge structures (van Kesteren et al., 2013; van Kesteren, Ruiter, Fernández, & Henson, 2012; Tse et al., 2011; Benchenane et al., 2010; van Kesteren, Fernández, Norris, & Hermans, 2010). However, mPFC did not show a significant subsequent memory effect. Subsequent memory for items was related to activity in the anterior medial-temporal lobe during encoding, consistent with several previous studies implicating the hippocampus (e.g., Wagner et al., 1998). Our subsequent memory effects also extended into the amygdala. This may be consistent with a role for the amygdala in item memory (Farovik, Place, Miller, & Eichenbaum, 2011; Kensinger, Addis, & Atapattu, 2011; Ranganath, 2010; Kensinger & Schacter, 2006) or with amygdala involvement in enhancing memory for items with affective salience (Hamann, Ely, Grafton, & Kilts, 1999) or intrinsic value as a reinforcer (Rolls, 2005). Unfortunately, we did not have enough statistical power to analyze subsequent memory effects separately in congruent, neutral, and incongruent items to address these possibilities.

The recollection of autobiographical information has been associated with a network of brain regions. Although many posterior regions have a hypothesized functional role within this network (e.g., Schacter et al., 2012; Hassabis & Maguire, 2009; Byrne et al., 2007; Cabeza & St Jacques, 2007), the mPFC has received somewhat less attention. AMs tend to be highly personal and value-laden. For example, we are more likely to remember the experience of having a cup of hot tea after walking outdoors for hours on a cold winter day than having a cup of tea on an ordinary afternoon. Given its association with value in decision-making and with the value afforded by imagined scenarios in this study and related studies (Benoit et al., 2014; Gross et al., 2014; Winecoff et al., 2013; Nieuwenhuis & Takashima, 2011), mPFC activity may reflect the value of recollected information (see also D’Argembeau, 2013). This is perhaps one reason why mPFC is typically not seen in more traditional episodic memory tasks, such as word recognition, where memory for such items may be high, but little value is associated with the retrieved items. Indeed, the subjective value associated with items may be one critical difference between typical autobiographical and episodic memory tasks.

To conclude, we have developed a new paradigm for looking at the interaction of imagery and value. We have validated it behaviorally via subjective value ratings and subsequent memory effects. Supporting our hypothesis, we found activity in the mPFC corresponding to the subjective value that an item is afforded by the imagined scenario. This suggests an extension of the well-known role of mPFC in representing value during decision-making and offers a potential explanation of its involvement in imagery and AM retrieval.

## Figures and Tables

**Table 1:** Percentage of Subjective Values of Items during Imagery (−3 to +3), according to Whether They Were Imagined in a Congruent, Incongruent, or Neutral State of Need, Controlling for Baseline Value (Value during Imagery—Everyday Value)

	*−3*	*−2*	*−1*	*0*	*+1*	*+2*	*+3*
Congruent	2.36%	5.20%	17.66%	35.10%	23.27%	11.95%	4.45%
Incongruent	6.63%	18.64%	34.98%	27.31%	8.93%	2.94%	0.58%
Neutral	6.35%	18.03%	30.90%	28.32%	11.43%	4.97%	0.00%

**Table 2:** Percentage of Hits, Misses, False Alarms, and Correct Rejections across Confidence Ratings (1–4) in the Old–New Item Recognition Task

	*Hit*	*Miss*	*False Alarm*	*Correct Rejection*
1	3.9	16.7	17.0	10.4
2	14.1	19.8	29.1	15.3
3	20.1	26.8	29.1	23.5
4	61.9	36.7	24.8	50.7

**Table 3:** Whole-brain fMRI Analysis Results

*Region*	*Cluster Size*	*x*	*y*	*z*	*Peak Z Score*
*The Subjective Value of Items in Imagery*					
Right mPFC	18	9	57	12	3.98*
Anterior cingulate	6	0	27	−9	3.1
Left ventral mPFC	8	−12	45	3	3.63
*Imagine States of Need > Imagine Neutral State*					
Left insula	6	−39	−6	−3	3.27
Right insula	1	45	15	3	3.15
*Congruent > Incongruent*					
Caudate	21	3	9	6	3.6
*Subsequent Memory Effect*					
Left hippocampus	3	−21	−12	−18	3.33
Right amygdala	2	33	−3	−30	3.27

All peaks reached an uncorrected significant level of *p* = .001.

* *p* value was <.05 at the cluster level with SVC.
